# Control Framework for Dexterous Manipulation Using Dynamic Visual Servoing and Tactile Sensors' Feedback

**DOI:** 10.3390/s140101787

**Published:** 2014-01-21

**Authors:** Carlos A. Jara, Jorge Pomares, Francisco A. Candelas, Fernando Torres

**Affiliations:** Physics, Systems Engineering and Signal Theory Department, University of Alicante, San Vicente del Raspeig, Alicante 03690, Spain; E-Mails: jpomares@ua.es (J.P.); francisco.candelas@ua.es (F.A.C.); fernando.torres@ua.es (F.T.)

**Keywords:** dexterous manipulation, dynamic control, tactile sensors, visual servoing

## Abstract

Tactile sensors play an important role in robotics manipulation to perform dexterous and complex tasks. This paper presents a novel control framework to perform dexterous manipulation with multi-fingered robotic hands using feedback data from tactile and visual sensors. This control framework permits the definition of new visual controllers which allow the path tracking of the object motion taking into account both the dynamics model of the robot hand and the grasping force of the fingertips under a hybrid control scheme. In addition, the proposed general method employs optimal control to obtain the desired behaviour in the joint space of the fingers based on an indicated cost function which determines how the control effort is distributed over the joints of the robotic hand. Finally, authors show experimental verifications on a real robotic manipulation system for some of the controllers derived from the control framework.

## 1. Introduction

Multi-fingered robotic hands allow both the execution of robust grasping tasks and dexterous manipulation. These are two of the skills that human beings have: dexterity and anthropomorphism [1]. These features enable multi-fingered hands to be controlled not only for holding the object with a firm grasp, but also for generating trajectories of the object with the movements of the fingers. This last form of motion control of the grasped object is usually known as “in-hand manipulation” or “dexterous manipulation”. In these cases, the kinematic redundancy of the fingers is used to change the object from an initial to a final configuration, while maintaining fingertip contacts [2,3]. Therefore, fingertip force control plays a very important role for dexterous manipulation in order to give the object the desired motion. For that end, tactile sensors are usually employed to measure the force exerted by the fingertips in order to maintain a desired value and to compute the contact points. This paper presents a new control framework to perform in-hand manipulation with multi-fingered robotic hands using feedback data from tactile and visual sensors.

Tactile sensors share a common property in robotics: they analyze the direct contact between the robot and the objects of the environment in order to adapt the robot's reaction to the manipulated object. Tactile information is processed according to two different aims: object identification and manipulation control. On the one hand, the properties of the objects extracted from the robot's tactile sensors can be used to categorize the objects into different classes. On the other hand, the measurements obtained from the tactile sensors can also be applied to control the interaction force [4]. In this paper, a hybrid scheme is proposed for manipulation control which takes into account both the robot hand dynamic model and the fingertips' forces.

Several approaches to solve the motion planning associated with dexterous manipulation problem have been proposed during the last decade. They have been mainly focused in three specific lines: graph representations [5–7], probabilistic trees [8,9] and hybrid control schemes [10,11]. Some of the previous planners (such as [5,6]) do not take into account the contact forces which are applied to the object and they are usually only based on the maintenance of the geometric contact between the surfaces of the fingers and the object (using surface models [12,13]), kinematic constraints and/or using manipulability analysis [14]. Fingertip force control is usually employed for planning in-hand manipulation algorithms based on hybrid control schemes [11]. The main objective of these algorithms is to control the grasping force to a desired value (satisfying friction conditions) besides controlling the position of the object along a desired trajectory given in the Cartesian space [4]. None of these previous works employs visual and tactile feedback to perform the manipulation task taking into account the robotic hand dynamics. This paper proposes a control framework that allows the definition of new controllers where the manipulated object motion is controlled using visual and tactile servoing algorithms.

Visual servo control techniques [15] allow the guidance of a robotic system using visual information usually in two types of configuration: eye-in-hand (camera fixed to the robot end) and eye-to-hand (external camera). This type of control is frequently applied in robot positioning tasks for a wide range of applications [16,17]. In robotic manipulation, visual techniques are usually applied as only computer vision algorithms for grasping [18,19]. Classical techniques of visual servo control do not take into account the system dynamics because they assume that the robot is a perfect positioning device. This assumption is not appropriate when the robot executes fast and/or accurate movements such as dexterous manipulation with multi-fingered robotic hands. By means of direct or dynamic visual servoing, the internal control loop of servo motors is removed and the visual servo control is used to stabilize the robotic system [20]. This paper uses direct visual servoing in order to perform a visual tracking of the manipulated object and the guidance of the robotic manipulation system. Therefore, in contrast with other control schemes for dexterous manipulation [10–19], the proposed approach takes into account the dynamics model of the robot hand employing visual and tactile sensory information.

In addition, the proposed control approach is based on an optimal control framework. This approach is used to visual servo control a robotic hand during a manipulation task taking into account the hand dynamics. From that framework, several new controllers are derived, which offers a useful unification methodology for direct control any robotic manipulation system using visual and tactile sensors feedback, an approach which has not been implemented in previous research projects. The proposed approach considers the optimization of the motor signals or torques sent to the robotic hand during visual control tasks. Moreover, the general method presented in the paper is based on the formulation of the tracking problem in terms of constraints, which was suggested in [21] inspired by results from analytical dynamics with constrained motion.

Summarizing, the proposed optimal control framework, from which several new dynamic controllers can be derived, is considered as the main contribution of this paper in comparison with other previous approaches for dexterous manipulation [5–14]. In addition, another contribution of this framework is that it uses direct visual servoing to perform the visual tracking of the manipulated object taking into account the dynamics model of the robot hand. In this sense, the presented optimal framework provides a useful unification methodology for the direct control of any robotic manipulation system using visual and tactile sensors feedback.

The paper is organized as follows: Section 2 describes the system architecture of the robotic manipulation system. Afterwards, the kinematics and dynamics formulation of the overall robotic system are described in Section 3. Section 4 explains the theoretical concepts about the employed optimal controller. In Section 5, the image trajectory to be tracked by the robotic manipulation system is described as a task constrain. In Section 6, the general dynamic visual servoing framework and the required modifications to perform a hybrid force control are presented. In Section 7, some new controllers are derived from the optimal control framework. Section 8 describes the experimental results illustrating the proposed controllers. The final section reports some important conclusions.

## 2. System Architecture

**Figure 1.**
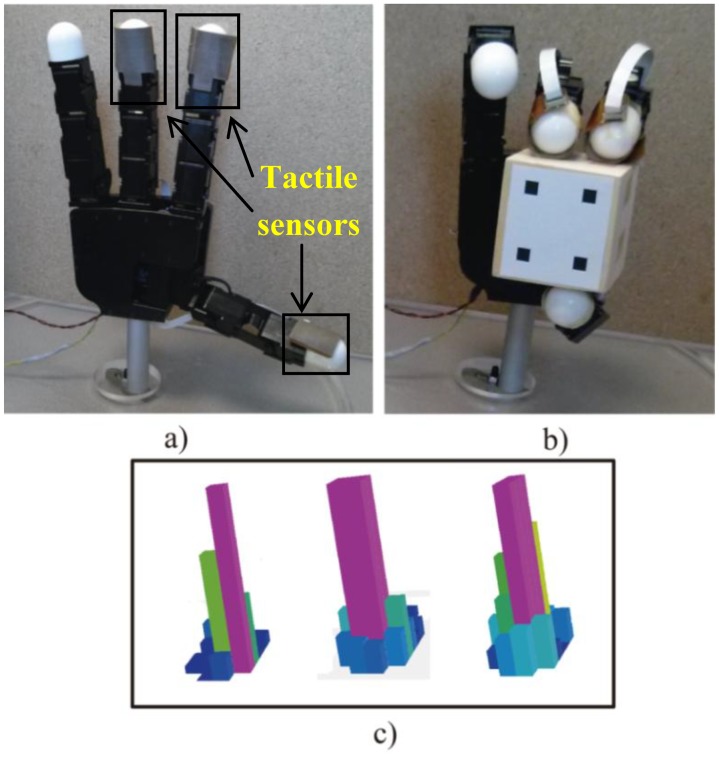
(**a**) Allegro hand with the tactile sensors installed in the fingertips' surface. (**b**) Allegro hand grasping the object to be manipulated. (**c**) Different 3D representation of the pressure measurements registered by the arrays of tactile sensors.

The robotic manipulation system is composed of the Allegro robotic hand (SimLab Co., Seoul, Korea) (see Figure 1a,b). This hand has four fingers and sixteen independent torque-controlled joints (four dof per each finger). This robotic hand has a lightweight and portable anthropomorphic design very suitable for low-cost dexterous manipulation in research. It is capable of holding up to 5 kg and it has support for real-time control and online simulation. In addition, a set of tactile sensors is employed as additional tool in the manipulation system (see Figure 1a). These sensors are installed in an extrinsic configuration [22] on the Allegro hand. These sensors are located at the three hand fingertips that will be used during the manipulation (furthermore, only the three last degrees of freedom of each finger will be controlled, performing a non-redundant system). The tactile sensors are pressure sensing arrays, type PPS RoboTouch (Pressure Profile Systems, Inc., Los Angeles, CA, USA). All these tactels can register pressure vales in the range 0–140 kPa with a frequency of 30 Hz and a sensitivity of 0.7 kPa. Figure 1c shows a 3D representation of the pressure measurements registered by these sensors during a manipulation task. The force exerted by the fingertip is computed using the pressure measurements of the tactile sensors. These measurements are multiplied by the area of each tactel, 25 mm^2^, so the forces are obtained. The mean of these forces is considered as the force applied by the fingertip. Moreover, the contact points are supposed to be in the tactel of maximum pressure exerted.

**Figure 2.**
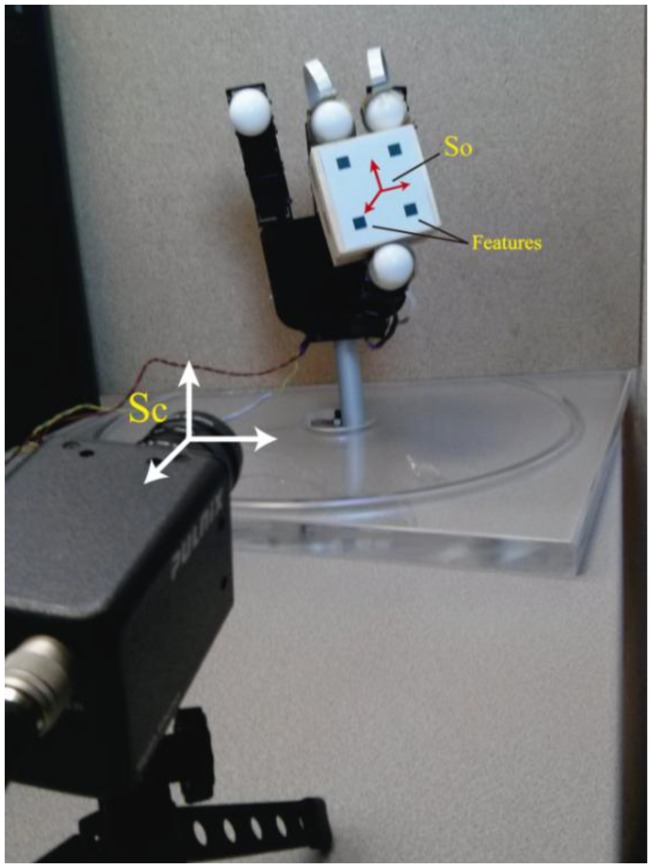
Experimental setup for dexterous manipulation (robotic hand, manipulated object, eye-to-hand camera and reference systems).

Figure 2 represents the experimental setup employed. For the visual servo control, a Gigabit Ethernet TM6740GEV camera (JAI-Pulnix, Shanghai, China) is used, which acquires 200 images every second with a resolution of 640 × 480 pixels. The camera is supposed to be previously calibrated and the camera intrinsic parameters are (u_0_, v_0_) = (298, 225) px, and (f_u_, f_v_) = (1,082.3, 1,073.7) px (position of the optical center (u_0_, v_0_) and the focal length in the x and y directions, respectively). The manipulated object has four marks which will be the extracted visual features.

## 3. Kinematics and Dynamics Formulation

### 3.1. Kinematics Equations

We consider the robotic hand as a set of k fingers with three degrees of freedom. Each finger holds an object considering contact points with friction and without slippage. In order to firmly grasp and manipulate the object, the grasp is considered to be an active form closure. Thus, each fingertip i is exerting a fingertip force **f**_Ci_ ∈ ℜ^3^ within the friction cone at the contact point. The grasping constrain between the robot and the object is done by the grasp matrix ${\text{J}}_{\text{G}}={\left[{\text{J}}_{\text{G}1}^{\text{T}}\ldots {\text{J}}_{\text{Gk}}^{\text{T}}\right]}^{\text{T}}\in {ℜ}^{3\text{k}\times 6}$ [23] which relates the contact forces **f**_C_ = [**f**_Cl_^T^ … **f**_Ck_^T^] ^T^ at the fingertips to the resultant force and moment **τ**_o_ ∈ ℜ^6^ on the object:

(1) $$\tau_{\text{o}}={\text{J}}_{\text{G}}\cdot {\text{f}}_{\text{C}}$$

where **f**_C_ and **τ**_o_ are both expressed in the object coordinate frame S_0_ fixed to the object mass center. This equation derives in the kinematics relation between velocity of the object **ẋ**_o_ ∈ ℜ^6^ and velocity of the contact point **v**_Ci_ ∈ ℜ^6^:

(2) $${\text{v}}_{\text{Ci}}={\text{J}}_{\text{Gi}}\cdot {\dot{\text{x}}}_{\text{o}}$$

where **x**_o_ denotes the position and orientation of the object in the contact point from S_0_. Extending Equation (2) for all the contact points and considering the object velocity with respect the camera coordinate frame, the following expression is obtained:

(3) $${\text{v}}_{\text{C}}={\text{J}}_{\text{GC}}\cdot {\dot{\text{x}}}_{\text{o}}^{\text{C}}$$

where **v**_C_ = [**v**_Cl_^T^ … **v**_Ck_^T^] ^T^ is the vector which contains all the contact points velocities.

As Figure 2 shows, a camera is fixed at the workspace in an eye-to-hand configuration in order to observe a set of features located at the surface of the manipulated object. The vector **s** = [*f*_1_*_x_*, *f*_1_*_y_*, *f*_2_*_x_*, *f*_2_*_y_*, …, *f_nx_*, *f_ny_*]^T^ ∈ ℜ^2n^ defines the image coordinates of the extracted features. From the interaction matrix **L**_s_(**s**) [24] it can be obtained the following relation:

(4) $${\dot{\text{s}}=\text{L}}_{\text{s}}(\text{s})\cdot {\dot{\text{x}}}_{\text{o}}^{\text{C}}$$

which relates the image information rate of change **ṡ** and object features velocity in the 3D space with respect the camera coordinate frame, ${\dot{\text{x}}}_{\text{o}}^{\text{C}}$.

The finger Jacobian denoted by **J**_Hi_ ∈ ℜ^3×3^ relates the joint velocities of the i^th^ finger (**q̇** ∈ ℜ^3^) with the fingertip velocities (**v**_Fi_ ∈ ℜ^3^) referenced in camera coordinate frame S_C_:

(5) $${\text{v}}_{\text{Fi}}={\text{J}}_{\text{Hi}}\cdot {\dot{\text{q}}}_{\text{i}}$$

This finger Jacobian can be easily obtained from the typical robot Jacobian matrix and applying the known mapping from robot coordinate frame and camera coordinate frame S_C_. Extending Equation (5) to all the k fingers of the robotic hand, this relation yields:

(6) $${\text{v}}_{\text{F}}={\text{J}}_{\text{H}}\cdot \dot{\text{q}}$$

Where ${\text{v}}_{\text{F}}={\left[{\text{v}}_{\text{F}1}^{\text{F}},\ldots ,{\text{v}}_{\text{Fk}}^{\text{T}}\right]}^{\text{T}}$ is the vector which contains all the fingertip velocities, **q̇** = [**q̇**_1_^T^ … **q̇**_k_^T^]^T^ represents the joint velocities of the robot hand and **J**_H_ = diag[**J**_H1_ … **J**_HK_] is the robot hand Jacobian which relates joint velocities and fingertip velocities measured from camera coordinate frame S_C_. If there is no slippage between the fingertips and the object, it can be considered that **v**_C_ = **v**_F_. Applying these equalities in Equations (3) and (6), we can obtain the main kinematic constrain in a robotic manipulation system:

(7) $${\text{J}}_{\text{GC}}\cdot {\dot{\text{x}}}_{\text{o}}^{\text{C}}{=\text{J}}_{\text{H}}\cdot \dot{\text{q}}$$

which relates object velocity from S_C_ with the finger joint velocities **q̇**. This equation must be accomplished in order to maintain the contact points fixed. From this kinematic constrain, we can get a relation between image space and joint space in a robotic manipulation system by using the interaction matrix described in Equation (4). This relation can be expressed with the following equation:

(8) $${\text{J}}_{\text{GC}}\cdot {{\text{L}}_{\text{s}}}^{+}\cdot \dot{\text{s}}={\text{J}}_{\text{H}}\cdot \dot{\text{q}}$$

where **L**_s_^+^ is the pseudo-inverse of the interaction matrix. From this equation, it can be obtained the joint velocities depending on the image features rate of change:

(9) $$\dot{\text{q}}={{\text{J}}_{\text{H}}}^{+}\cdot {\text{J}}_{\text{GC}}\cdot {{\text{L}}_{\text{s}}}^{+}\cdot {\dot{\text{s}}=\text{J}}_{\text{T}}\cdot \dot{\text{s}}$$

Where **J**_T_ is the Jacobian matrix mapping from joint space to image space in this robotic manipulation system.

### 3.2. Manipulation System Dynamics

The dynamic model of the manipulation system can be divided into the dynamics description both the grasped object and the multi-fingered hand with the contact forces constrain. In this subsection, both dynamics equations will be given.

The motion equation for the object based on the simple case of moving it in free space without any external force, can be described as:

(10) $${\text{M}}_{\text{o}}\cdot {\ddot{\text{x}}}_{\text{s}}{+\text{C}}_{\text{o}}{+\text{g}}_{\text{o}}{=\tau }_{\text{o}}$$

Where M_o_ ∈ ℜ^6×6^ is the inertia matrix of the object, **C**_o_ ∈ ℜ^6^ is the centrifugal and Coriolis vector, **g**_o_ ∈ ℜ^6^ is the gravitational force and **τ**_o_ ∈ ℜ^6^ is the resultant force applied by the fingers. The variable **ẍ**_s_ ∈ ℜ^6^ is the desired object acceleration.

With regard to the multi-fingered hand, we can assume its dynamics as the set of serial three-link rigid mechanism which correspond to the fingers. In this case, the dynamics equation of finger i can be described as:

(11) $${\text{M}}_{\text{Fi}}({\text{q}}_{\text{i}})\cdot {\ddot{\text{q}}}_{\text{i}}+{\text{C}}_{\text{Fi}}({\text{q}}_{\text{i}},{\dot{\text{q}}}_{\text{i}})+{\text{g}}_{\text{Fi}}({\text{q}}_{\text{i}})+{\text{J}}_{\text{i}}^{\text{T}}\cdot {\text{f}}_{\text{Ci}}=\tau_{\text{i}}$$

Where **q**_i_ ∈ ℜ^3×1^, **q̇**_i_ ∈ ℜ^3×1^ and **q̈**_i_ ∈ ℜ^3×1^ are the vectors of generalized joint coordinates, joint velocities and joint accelerations of the finger i. Moreover, **M**_Fi_ ∈ ℜ^3×3^ is the symmetric positive definite finger inertia matrix, **C**_Fi_ ∈ ℜ^3×1^ and **g**_Fi_ ∈ ℜ^3×1^ both denote the vector of centripetal and Coriolis forces and the gravitational force of the finger i, respectively. In addition, **τ**_i_ ∈ ℜ^3×1^ represents the applied motor commands (*i.e.*, joint torques) in the finger i and **f**_Ci_ ∈ ℜ^3×1^ is the contact forces exerted by the finger i at its contact point. Combining Equation (11) for all k the fingers, we obtain the dynamics model of a multi-fingered robotic hand (for the sake of clarity the time and joint dependences are not indicated):

(12) $${\text{M}}_{\text{H}}\cdot \ddot{\text{q}}+{\text{C}}_{\text{H}}+{\text{g}}_{\text{H}}+{\text{J}}_{\text{H}}^{\text{T}}\cdot {\text{f}}_{\text{C}}=\tau$$

Where **M**_H_ = diag[**M**_F1_…**M**_Fk_], **C**_H_ = col[**C**_F1_…**C**_Fk_], **g**_H_ = col[**g**_F1_…**g**_Fk_], **τ** = col[**τ**_1_…**τ**_k_], **f**_C_ = col[**f**_C1_…**f**_Ck_] are the composition of the matrices and vector for the whole system. The term ${\text{J}}_{\text{H}}^{\text{T}}\cdot {\text{f}}_{\text{C}}$ represents the torques derived from the kinematic constrain in a robotic manipulation system represented by Equation (7).

## 4. Optimal Control Framework

As stated, Equation (12) represents the dynamics of a multi-fingered robot hand in a robotic manipulation system. If we do not take into account the kinematic constraint between hand-object (Equation (7)), the dynamic model of the multi-fingered robot hand becomes the following expression:

(13) $${\text{M}}_{\text{H}}\cdot \ddot{\text{q}}+{\text{C}}_{\text{H}}+{\text{g}}_{\text{H}}=\tau$$

where **τ** ∈ ℜ^3k×1^ represents the applied motor commands at the joints' fingers. In order to simplify this equation, we can write the robot hand dynamics as follows:

(14) $${\text{M}}_{\text{H}}\ddot{\text{q}}=\tau +{\text{F}}_{\text{cg}}$$

where **F**_cg_ = −**C**_H_ − **g**_H_.

The dynamic model of a serial-link robot has been used in different approaches to control a robotic system for tracking [25]. Following this idea, the approach proposed in [21] gave a new perspective about tracking based on optimal control for nonlinear mechanical systems. This approach will be used in this paper in order to perform a visual tracking of the grasped object in a robotic manipulation system.

Basically, the control approach suggested by [21] supposes a system with m constraints given by:

(15) $$\varphi (\text{q},\dot{\text{q}},\text{t})=0$$

This equation may contain holonomic and/or non-holonomic constraints, and represents the task for the robotic system to be described in form of m constraints description. Differentiating these constraints with respect to time (assuming that *φ* is sufficiently smooth), the following equation can be obtained:

(16) $$\text{A}(\text{q},\dot{\text{q}},\text{t})\ddot{\text{q}}=\text{b}(\text{q},\dot{\text{q}},\text{t})$$

Where **A**(**q**, **q̇**, t) ∈ ℜ^m×3k^ and **b**(**q**, **q̇**, t) ∈ ℜ^m×1^ are both matrix and vector obtained by differentiating the set of relations which satisfy the constrains represented by Equation (15). The goal of the optimal controller is to minimize the control torques of the mechanical system while performing a specific task taking into account the following function cost:

(17) $$\Omega (\text{t})=\tau^{\text{T}}\text{W}(\text{t})\tau$$

where **W**(t) is a time-dependent weight matrix. According to [21], the function control that minimizes **Ω**(t) of the mechanical system based on Equation (14) while performing the task described in Equation (16) is given by:

(18) $$\tau ={\text{W}}^{-1/2}{\left({{\text{AM}}_{\text{H}}}^{-1}{\text{W}}^{-1/2}\right)}^{+}\cdot \left(\text{b}-{{\text{AM}}_{\text{H}}}^{-1}{\text{F}}_{\text{cg}}\right)$$

where **M**_H_ is the inertia matrix of the robotic system, in this case of the multi-fingered robot hand, and the symbol + denotes the pseudo-inverse for a general matrix. As it can be seen in Equation (18), the matrix **W** is an important depending variable in the control law and determines how the control effort is distributed over the joints.

Although the optimal control is based on the robotic hand dynamic model of Equation (14), it can be applied to the robotic manipulation system model presented in Equation (12). This fact can be done considering the kinematic constrain between the robotic hand and the object (term ${\text{J}}_{\text{H}}^{\text{T}}\cdot {\text{f}}_{\text{C}}$ of Equation (12)) as a desired value in a hybrid control force of the robotic manipulation system (see Section 6).

## 5. Constrained Task Description in the Robotic Manipulation System

The main objective of the robotic manipulation system proposed is to control the grasping force to a desired value such that the friction condition is satisfied besides controlling the position of the object along a desired trajectory in the image space. Therefore, the task description as constraint is given by the following equation in the image space:

(19) $$({\ddot{\text{s}}}_{\text{d}}-\ddot{\text{s}})+{\text{K}}_{\text{D}}({\dot{\text{s}}}_{\text{d}}-\dot{\text{s}})+{\text{K}}_{\text{P}}({\text{s}}_{\text{d}}-\text{s})=0$$

Where **s̈**_d_, **ṡ**_d_ and **s**_d_ are the desired image space accelerations, velocities and positions, respectively. **K**_P_ and **K**_D_ are proportional and derivative gain matrices, respectively. This equation can be expressed with regard to image error in the following way:

(20) $${\ddot{\text{s}}}_{\text{d}}+{\text{K}}_{\text{D}}{\dot{\text{e}}}_{\text{s}}+{\text{K}}_{\text{P}}{\text{e}}_{\text{s}}={\ddot{\text{s}}}_{\text{r}}$$

Where **e**_s_ and **ė**_s_ are the image error and the time derivative of the error respectively. The variable **s̈**_r_ denotes the reference image accelerations of our image space based controller. This reference control is related with joint accelerations by differentiating to the time Equation (9) after solving image velocities:

(21) $${\dot{\text{s}}}_{\text{r}}={{\text{J}}_{\text{T}}}^{+}\dot{\text{q}}$$

(22) $${\ddot{\text{s}}}_{\text{r}}={{\text{J}}_{\text{T}}}^{+}\ddot{\text{q}}+{{\dot{\text{J}}}_{\text{T}}}^{+}\dot{\text{q}}$$

where **J**_T_^+^ is the pseudo-inverse of the Jacobian **J**_T_. Equation (21) describes the relation between the reference control in the image space and the joint variables of the robot hand. We assume that **J**_T_ is continuously differentiable with respect to joint coordinates **q** and that it has full rank. Using this last expression and replacing it into Equation (20), it is obtained the following expression:

(23) $${\ddot{\text{s}}}_{\text{d}}+{\text{K}}_{\text{D}}{\dot{\text{e}}}_{\text{s}}+{\text{K}}_{\text{P}}{\text{e}}_{\text{s}}-{{\dot{\text{J}}}_{\text{T}}}^{+}\dot{\text{q}}={{\text{J}}_{\text{T}}}^{+}\ddot{\text{q}}$$

From this equation, it can be possible to express the image tracking or task in the form of the constraints description of Equation (16) with:

(24) $$\begin{array}{l} \text{A}={{\text{J}}_{\text{T}}}^{+}\\ \text{b}={\ddot{\text{s}}}_{\text{d}}+{\text{K}}_{\text{D}}{\dot{\text{e}}}_{\text{s}}+{\text{K}}_{\text{P}}{\text{e}}_{\text{s}}-{{\dot{\text{J}}}_{\text{T}}}^{+}\dot{\text{q}} \end{array}$$

With this definition of **A** and **b**, the optimal control will minimize the torques of robot hand while performing a tracking in the image space. With regard to the manipulated object, the motion equation is determined by Equation (10). Using the relation described by Equation (1), the object motion yields:

(25) $${\text{M}}_{\text{o}}\cdot {\ddot{\text{x}}}_{\text{s}}+{\text{C}}_{\text{o}}+{\text{g}}_{\text{o}}={\text{J}}_{\text{G}}\cdot {\text{f}}_{\text{C}}$$

where **ẍ**_s_ is the object acceleration imposed by the reference controller. By differentiating with respect to the time Equation (4), we can relate the object acceleration and the reference control **s̈**_r_:

(26) $${\ddot{\text{s}}}_{\text{r}}={\text{L}}_{\text{s}}\cdot {\ddot{\text{x}}}_{\text{s}}+{\dot{\text{L}}}_{\text{s}}\cdot {\dot{\text{x}}}_{\text{s}}$$

Solving the motion acceleration of the object from Equation (26) and replacing it in Equation (25), the following expression which relates the reference image acceleration and the desired behaviour of the object taken into account the contact forces is obtained:

(27) $${\text{M}}_{\text{o}}\cdot {{\text{L}}_{\text{s}}}^{+}\cdot \left[{\ddot{\text{s}}}_{\text{r}}-{\dot{\text{L}}}_{\text{s}}\cdot {\dot{\text{x}}}_{\text{s}}\right]+{\text{C}}_{\text{o}}+{\text{g}}_{\text{o}}={\text{J}}_{\text{G}}\cdot {\text{f}}_{\text{C}}$$

## 6. Dynamic Visual Control Framework

This section describes a new optimal control framework for a robotic manipulation system using direct visual servoing. This control framework is based on the task description in the image space explained in the section above.

### 6.1. Visual Controller

As stated, the control function that minimizes the motor signals of a robot hand while performing the task described in Equation (14) is given by Equation (18). Replacing the variables concerned on the task description, **A** and **b** from Equation (24), the control law is set by:

(28) $$\tau ={\text{W}}^{-1/2}{\left({{\text{J}}_{\text{T}}}^{+}{{\text{M}}_{\text{H}}}^{-1}{\text{W}}^{-1/2}\right)}^{+}\cdot \left({\ddot{\text{s}}}_{\text{d}}+{\text{K}}_{\text{D}}{\dot{\text{e}}}_{\text{s}}+{\text{K}}_{\text{P}}{\text{e}}_{\text{s}}-{{\dot{\text{J}}}_{\text{T}}}^{+}\dot{\text{q}}-{{\text{J}}_{\text{T}}}^{+}{{\text{M}}_{\text{H}}}^{-1}{\text{F}}_{\text{cg}}\right)$$

As it can be seen, the control law (28) depends implicitly on the weighting matrix **W** and different values of this matrix can simplify the product (**J**_T_^+^**M**_H_^−1^**W**_T_^−1/2^)^+^ and consequently, the control law. Different control laws will be presented in the next section with different values of **W**.

In order to demonstrate the stability of the control law, the closed loop is computed from Equations (14) and (28) as:

(29) $$\text{M}\cdot \ddot{\text{q}}-{\text{F}}_{\text{cg}}={\text{W}}^{-1/2}{\left({{\text{J}}_{\text{T}}}^{+}{{\text{M}}_{\text{H}}}^{-1}{\text{W}}^{-1/2}\right)}^{+}\cdot \left({\ddot{\text{s}}}_{\text{d}}+{\text{K}}_{\text{D}}{\dot{\text{e}}}_{\text{s}}+{\text{K}}_{\text{P}}{\text{e}}_{\text{s}}-{{\dot{\text{J}}}_{\text{T}}}^{+}\dot{\text{q}}-{{\text{J}}_{\text{T}}}^{+}{{\text{M}}_{\text{H}}}^{-1}{\text{F}}_{\text{cg}}\right)$$

This Equation can be simplified by pre-multiplying its left and right side by the term (**J**_T_^+^**M**_H_^−1^**W**_T_^−1/2^)**W**^1/2^:

(30) $${{\text{J}}_{\text{T}}}^{+}\ddot{\text{q}}={\ddot{\text{s}}}_{\text{d}}+{\text{K}}_{\text{D}}{\dot{\text{e}}}_{\text{s}}+{\text{K}}_{\text{P}}{\text{e}}_{\text{s}}-{{\dot{\text{J}}}_{\text{T}}}^{+}\dot{\text{q}}$$

Using Equations (20) and (22), from Equation (30) it can be concluded that:

(31) $${\ddot{\text{e}}}_{\text{s}}=-{\text{K}}_{\text{D}}{\dot{\text{e}}}_{\text{s}}+{\text{K}}_{\text{P}}{\text{e}}_{\text{s}}$$

Therefore, when **J**_T_^+^is full rank, an asymptotic tracking is achieved. This way, the convergence of the visual servo control law is demonstrated.

### 6.2. Force Fingertip Control

The manipulation of an object by a multi-fingered robot hand with fixed contact points alters the robot's dynamics with a generalized contact force **f**c acting on the end effector of the fingers (see Equation (12)). In this framework, dynamic control of a robotic manipulation system is considered as a hybrid control produced from the interaction between a robot mechanism (robotic hand) and the environment (objet to be manipulated). This way, in this paper an approach for intending both to control the desired position of the object using visual features and the desired contact force exerted by the fingertips is presented.

In order to incorporate a control which in practice keeps constant the contact forces without affecting the task achievement or image tracking, a modification of the control law defined in Equation (28) is performed. As it is shown in this equation, the task space is defined by the expression **W**^−1/2^(**J**_T_^+^**M**_H_^−1^**W**^−1/2^)^+^. Therefore, the term expressed as [**I** − **W**^−1/2^(**J**_T_^+^**M**_H_^−1^**W**^−1/2^)^+^ (**J**_T_^+^**M**_H_^−1^**W**^−1/2^)**W**^1/2^] · **τ**_1_ can be used to project the motor command **τ**_1_ onto the null space of the dynamic visual servo task. The fulfillment of the task is not affected by the choice of the control law **τ**_1_. Setting **τ**_1_ = **C**_H_ + **g**_H_ + **τ**_0_, the Coriolis, centrifugal and gravitational forces can be compensated. Using this modification, the control law yields as follows:

(32) $$\tau ={\text{W}}^{-1/2}{\left({{\text{J}}_{\text{T}}}^{+}{{\text{M}}_{\text{H}}}^{-1}{\text{W}}^{-1/2}\right)}^{+}\cdot \left({\ddot{\text{s}}}_{\text{d}}+{\text{K}}_{\text{D}}{\dot{\text{e}}}_{\text{s}}+{\text{K}}_{\text{P}}{\text{e}}_{\text{s}}-{{\dot{\text{J}}}_{\text{T}}}^{+}\dot{\text{q}}\right)+{\text{C}}_{\text{H}}+{\text{g}}_{\text{H}}+{\text{W}}^{-1/2}\cdot \left[\text{I}-{\left({{\text{J}}_{\text{T}}}^{+}{{\text{M}}_{\text{H}}}^{-1}{\text{W}}^{-1/2}\right)}^{+}\left({{\text{J}}_{\text{T}}}^{+}{{\text{M}}_{\text{H}}}^{-1}{\text{W}}^{-1/2}\right)\right]{\text{W}}^{1/2}\cdot \tau_{0}$$

where the joint control law **τ**_0_ works in the null space of the task defined by (**J**_T_^+^**M**_H_^−1^**W**^−1/2^)^+^. In this paper, the term **τ**_0_ represents an additional component of the final controller which is used for contact force stabilization. For this reason, this term is defined as follows:

(33) $$\tau_{0}={\text{J}}_{\text{H}}^{\text{T}}\cdot {\text{f}}_{\text{d}}$$

where **f**_d_ are the desired exerted contact forces of the fingertips which act in the null-space. Therefore, both the constraint imposed by the image tracking and the contact force can be set independently and accomplished with the control law presented in Equation (32).

## 7. Derived Visual Servo Controllers

One of the main contributions of the proposed framework is the possibility to generate different controllers to perform robotic manipulation tasks taking into account the robot dynamics and using visual-tactile information. Up to now, the control law has been written depending on the weighting matrix **W**. As stated, the choice of **W** plays an important role in the controller because it determines how the torques are distributed over the joints. In the next subsections, new control laws are obtained from the choice of different values of **W**.

### 7.1. Using W= $\text{W}\;={\text{M}}_{\text{H}}^{-2}$

Considering $\text{W}\;={\text{M}}_{\text{H}}^{-2}$ as weighting matrix, the control law from Equation (32) yields:

(34) $$\tau ={\text{M}}_{\text{H}}{\left({{\text{J}}_{\text{T}}}^{+}{{\text{M}}_{\text{H}}}^{-1}{\text{M}}_{\text{H}}\right)}^{+}\cdot \left({\ddot{\text{s}}}_{\text{d}}+{\text{K}}_{\text{D}}{\dot{\text{e}}}_{\text{s}}+{\text{K}}_{\text{P}}{\text{e}}_{\text{s}}-{{\dot{\text{J}}}_{\text{T}}}^{+}\dot{\text{q}}-{{\text{J}}_{\text{T}}}^{+}{{\text{M}}_{\text{H}}}^{-1}{\text{F}}_{\text{cg}}\right)+{\text{M}}_{\text{H}}\cdot \left[\text{I}-{\left({{\text{J}}_{\text{T}}}^{+}{{\text{M}}_{\text{H}}}^{-1}{\text{M}}_{\text{H}}\right)}^{+}\left({{\text{J}}_{\text{T}}}^{+}{{\text{M}}_{\text{H}}}^{-1}{\text{M}}_{\text{H}}\right)\right]\cdot {{\text{M}}_{\text{H}}}^{-1}\cdot {\text{J}}_{\text{H}}^{\text{T}}{\text{f}}_{\text{d}}$$

Simplifying this equation, the final control law gives the following expression:

(35) $$\tau ={\text{M}}_{\text{H}}{\text{J}}_{\text{T}}\left({\ddot{\text{s}}}_{\text{d}}+{\text{K}}_{\text{D}}{\dot{\text{e}}}_{\text{s}}+{\text{K}}_{\text{P}}{\text{e}}_{\text{s}}-{{\dot{\text{J}}}_{\text{T}}}^{+}\dot{\text{q}}\right)+{\text{C}}_{\text{H}}+{\text{g}}_{\text{H}}+{\text{M}}_{\text{H}}\cdot \left[\text{I}-{\text{J}}_{\text{T}}\cdot {{\text{J}}_{\text{T}}}^{+}\right]\cdot {{\text{M}}_{\text{H}}}^{-1}\cdot {\text{J}}_{\text{H}}^{\text{T}}{\text{f}}_{\text{d}}$$

As it can be seen in the Equation (35), this choice of the **W** matrix has allowed the decoupling of kinematics and dynamics of the robot hand in the control law. This is because the null-space term is based on **J**_T_, a matrix with only kinematic contain.

### 7.2. Using W= $\text{W}\;={\text{M}}_{\text{H}}^{-1}$

An important value for the control law due to its physical interpretation is $\text{W}\;={\text{M}}_{\text{H}}^{-1}$ since it is consistent with the principle of d'Alembert [25]. Using this value for **W**, the control law expressed in the Equation (32) results as follows:

(36) $$\tau ={{\text{M}}_{\text{H}}}^{1/2}{\left({{\text{J}}_{\text{T}}}^{+}{{\text{M}}_{\text{H}}}^{-1/2}\right)}^{+}\cdot \left({\ddot{\text{s}}}_{\text{d}}+{\text{K}}_{\text{D}}{\dot{\text{e}}}_{\text{s}}+{\text{K}}_{\text{P}}{\text{e}}_{\text{s}}-{{\dot{\text{J}}}_{\text{T}}}^{+}\dot{\text{q}}\right)+{\text{C}}_{\text{H}}+{\text{g}}_{\text{H}}+{{\text{M}}_{\text{H}}}^{1/2}\cdot \left[\text{I}-{\left({{\text{J}}_{\text{T}}}^{+}{{\text{M}}_{\text{H}}}^{-1/2}\right)}^{+}\left({{\text{J}}_{\text{T}}}^{+}{{\text{M}}_{\text{H}}}^{-1/2}\right)\right]\cdot {{\text{M}}_{\text{H}}}^{-1/2}\cdot {\text{J}}_{\text{H}}^{\text{T}}{\text{f}}_{\text{d}}$$

Applying the pseudo inverse as Q^+^ = Q^T^(Q˙Q^T^)^−1^ and simplifying this equation, the control law yields:

(37) $$\tau ={{\text{J}}_{\text{T}}}^{{+}^{\text{T}}}{\left({{\text{J}}_{\text{T}}}^{+}{{\text{M}}_{\text{H}}}^{-1}{{\text{J}}_{\text{T}}}^{{+}^{\text{T}}}\right)}^{-1}\cdot \left({\ddot{\text{s}}}_{\text{d}}+{\text{K}}_{\text{D}}{\dot{\text{e}}}_{\text{s}}+{\text{K}}_{\text{P}}{\text{e}}_{\text{s}}-{{\dot{\text{J}}}_{\text{T}}}^{+}\dot{\text{q}}\right)+{\text{C}}_{\text{H}}+{\text{g}}_{\text{H}}+{\text{M}}_{\text{H}}\cdot \left[\text{I}-{{\text{M}}_{\text{H}}}^{-1}{{\text{J}}_{\text{T}}}^{{+}^{\text{T}}}{\left({{\text{J}}_{\text{T}}}^{+}{{\text{M}}_{\text{H}}}^{-1}{{\text{J}}_{\text{T}}}^{{+}^{\text{T}}}\right)}^{-1}\right]\cdot {{\text{M}}_{\text{H}}}^{-1}\cdot {\text{J}}_{\text{H}}^{\text{T}}{\text{f}}_{\text{d}}$$

### 7.3. Using W= DM_H_^−2^

In this subsection, a new value of $\text{W}={\text{DM}}_{\text{H}}^{-2}$, where **D** is a diagonal positive matrix, will be employed. This matrix allows distributing the torques to the joints of the robot hand, therefore, large weights in this matrix causes small torques. Using this value for **W**, the controller from Equation (37) results as follows:

(38) $$\tau ={\left({{\text{DM}}_{\text{H}}}^{-2}\right)}^{-1/2}{\left({{\text{J}}_{\text{T}}}^{+}{{\text{M}}_{\text{H}}}^{-1}{\left({{\text{DM}}_{\text{H}}}^{-2}\right)}^{-1/2}\right)}^{+}\cdot \left({\ddot{\text{s}}}_{\text{d}}+{\text{K}}_{\text{D}}{\dot{\text{e}}}_{\text{s}}+{\text{K}}_{\text{P}}{\text{e}}_{\text{s}}-{{\dot{\text{J}}}_{\text{T}}}^{+}\dot{\text{q}}\right)+{\text{C}}_{\text{H}}+{\text{g}}_{\text{H}}+{\left({{\text{DM}}_{\text{H}}}^{-2}\right)}^{-1/2}\cdot \left[\text{I}-{\left({{\text{J}}_{\text{T}}}^{+}{{\text{M}}_{\text{H}}}^{-1}{\left({{\text{DM}}_{\text{H}}}^{-2}\right)}^{-1/2}\right)}^{+}\left({{\text{J}}_{\text{T}}}^{+}{{\text{M}}_{\text{H}}}^{-1}{\left({{\text{DM}}_{\text{H}}}^{-2}\right)}^{-1/2}\right)\right]\cdot {\left({{\text{DM}}_{\text{H}}}^{-2}\right)}^{-1/2}\cdot {\text{J}}_{\text{H}}^{\text{T}}{\text{f}}_{\text{d}}$$

Simplifying the equation as before, the control law yields:

(39) $$\tau ={\text{M}}_{\text{H}}{\text{D}}^{-1}{\text{J}}_{\text{T}}^{{\text{T}}^{+}}\cdot {\left({{\text{J}}_{\text{T}}}^{+}{\text{D}}^{-1}{\text{J}}_{\text{T}}^{{\text{T}}^{+}}\right)}^{-1}\cdot \left({\ddot{\text{s}}}_{\text{d}}+{\text{K}}_{\text{D}}{\dot{\text{e}}}_{\text{s}}+{\text{K}}_{\text{P}}{\text{e}}_{\text{s}}-{{\dot{\text{J}}}_{\text{T}}}^{+}\dot{\text{q}}\right)+{\text{C}}_{\text{H}}+{\text{g}}_{\text{H}}+{\text{M}}_{\text{H}}\text{D}\cdot \left[\text{I}-{\text{D}}^{-1}{\text{J}}_{\text{T}}^{{\text{T}}^{+}}{\left({{\text{J}}_{\text{T}}}^{+}{\text{M}}^{-1}{\text{J}}_{\text{T}}^{{\text{T}}^{+}}\right)}^{-1}{{\text{J}}_{\text{T}}}^{+}\right]\cdot {\text{M}}_{\text{H}}{\text{D}}^{-1}\cdot {\text{J}}_{\text{H}}^{\text{T}}{\text{f}}_{\text{d}}$$

## 8. Experimental Results

In the previous section, three visual controllers have been obtained using the proposed control framework. In this section, different results are described in order to evaluate the obtained controllers during manipulation tasks using the system architecture presented in Section 2. To do this, four visual features are extracted from the grasped object by using the eye-to-hand camera system. It is assumed that all the visual features are visible during the manipulation experiment. As stated, only three fingers of the robot hand and its three last degrees of freedom are employed in the manipulation task.

### 8.1. Behavior in the Joint and Image Spaces

**Figure 3.**
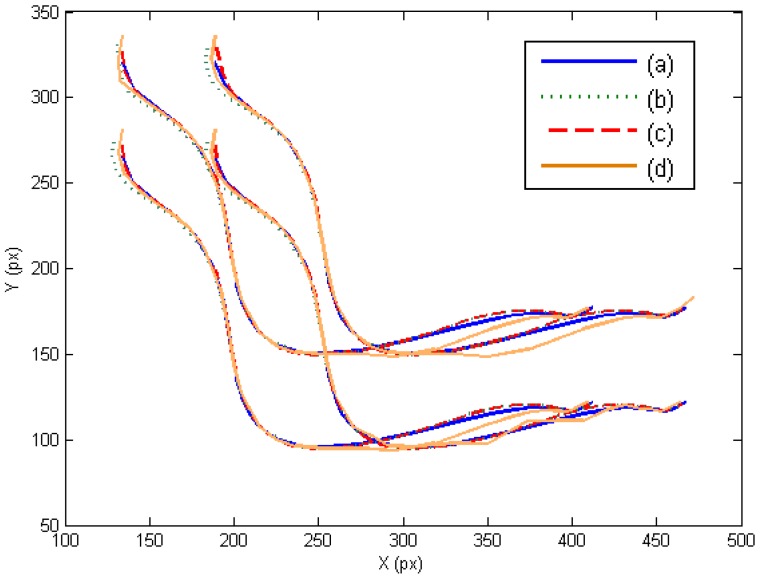
Image trajectories obtained during the first set of experiments. (**a**) Desired image trajectory. (**b**) **W** = **M**^−2^. (**c**) **W** = **DM**^−2^. (**d**) **W** = **M**^−1^.

In this section, the three controllers derived in Section 7 are evaluated in the joint and image space. The presented experiments consist of a manipulation task where the extracted image features must track the desired image trajectory represented in Figure 3a. This trajectory has been previously defined like a time-dependent function. In this case, the external eye-to-hand camera is located at a distance of 50 cm observing the trajectory described by the four points located at the object. Figure 3b,c,d represents the obtained image trajectory when **W** = **M**^−2^, **W** = **DM**^−2^ and **W** = **M**^−1^, respectively. As this last figure shows, the tracking is correctly developed in the image space and the image error remains low (Table 1).

**Figure 4.**
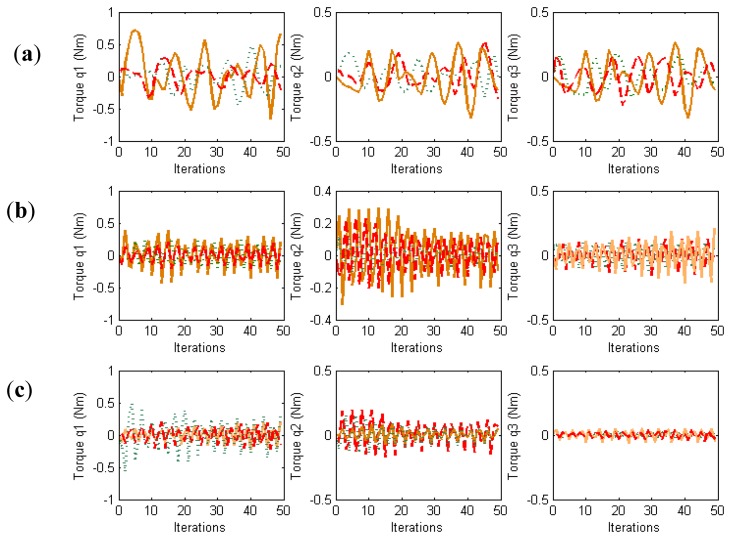
Torques obtained during the first set of experiments. (**a**) First finger. (**b**) Second finger. (**c**) Third finger. For each figure the torques obtained by the controllers indicated in the legend of Figure 3 are represented.

In order to evaluate the behavior in the joint space during these manipulation experiments, the obtained torques are represented in Figure 4. For each joint of each finger, the torques when **W** = **M**^−2^, **W** = **DM**^−2^ and **W** = **M**^−1^ are indicated. When **W** = **DM**^−2^, the value of **D** allows us to indicate which joints will support high loads. In this experiment, the weight value corresponding to the first joint is twice the weight corresponding to the second and third joints. Comparing the torques of the first joints (first column in Figure 4) and the torques of the second joints (second column in Figure 4), it can be observed that when **W** = **DM**^−2^ (in red) lower torques in the first joints are obtained. Therefore, this diagonal matrix can be employed to distribute the torques and to diminish the effort in the desired joints. When **W** = **M**^−1^ a correct image tracking is also observed (see Figure 2), however a more oscillating behaviour is obtained in the joint space.

**Figure 5.**
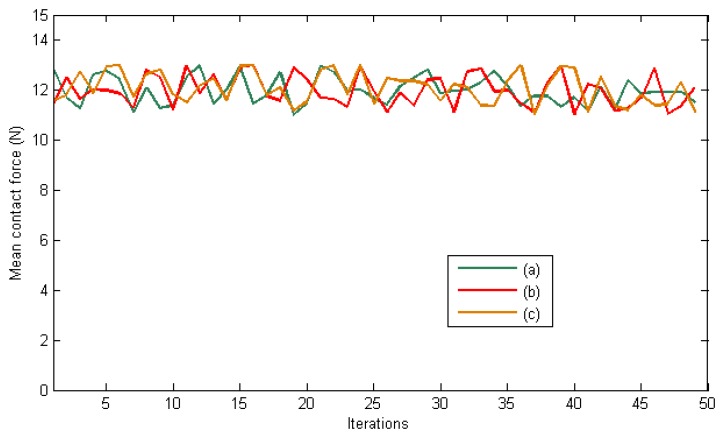
Mean total contact force during the first set of experiments. (**a**) **W** = **M**^−2^. (**b**) **W** = **DM**^−2^. (**c**) **W** = **M**^−1^.

**Figure 6.**
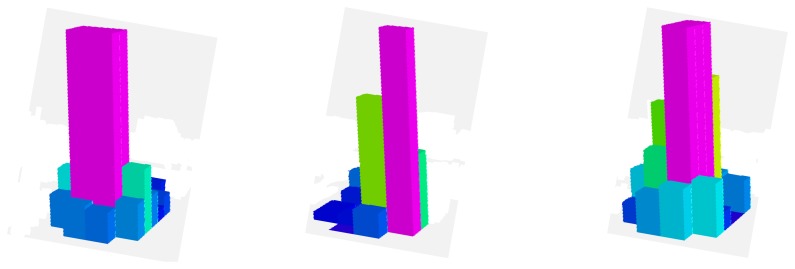
Distribution of the pressure measurements registered by the arrays of tactile sensors of the three fingers in an intermediate iteration during the manipulation task (first task).

The desired contact forces for the fingertips are regulated to 12 N during the experiment. Figure 5 represents the mean total contact force during the experiment. Furthermore, Figure 6 indicates the distribution of the pressure measurements registered by the arrays of tactile sensors of the three fingers in an intermediate iteration during the manipulation task. The force contact error also remains low and the mean error when **W** = **M**^−2^, **W** = **DM**^−2^ and **W** = **M**^−1^ are 0.87 N, 1.01 N and 0.91 N respectively.

### 8.2. Behavior in the 3D Space

**Figure 7.**
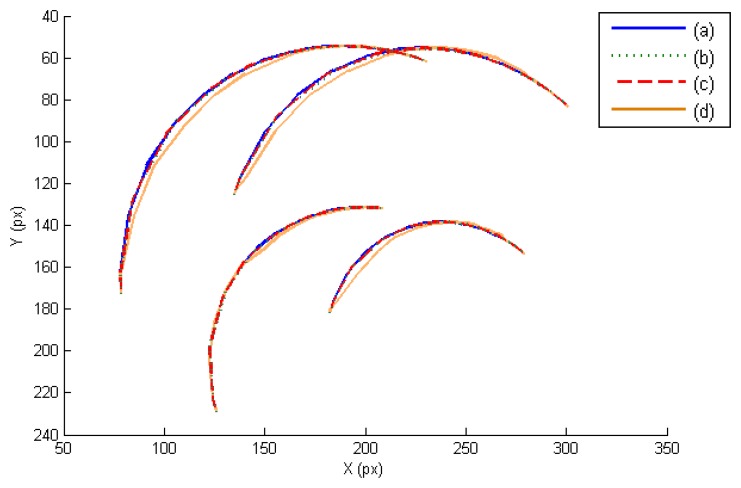
Image trajectories obtained during the second set of experiments. (**a**) Desired image trajectory. (**b**) **W** = **M**^−2^. (**c**) **W** = **DM**^−2^. (**d**) **W** = **M**^−1^.

**Figure 8.**
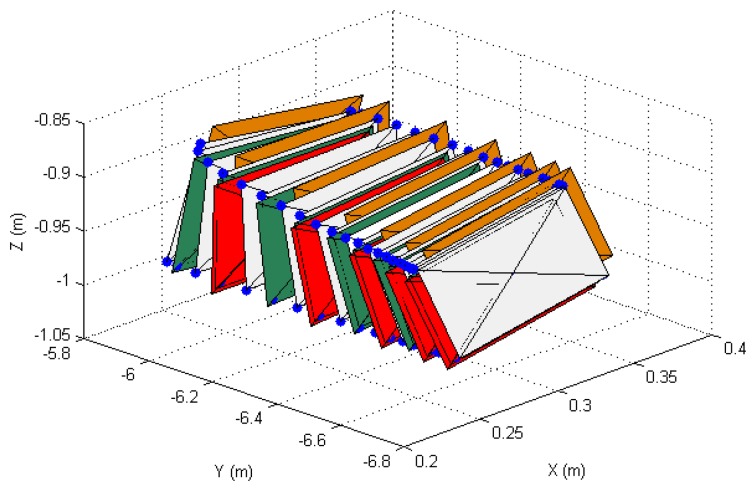
3D trajectories of the manipulated object obtained during the second set of experiments.

In order to show the 3D behavior of the proposed controllers, this section presents a manipulation task where the grasped object must perform a rotation while the robot is doing a displacement. The image trajectories described by the four extracted marks using the three controllers are represented in Figure 7. As it was noted in the previous section, the three controllers guide correctly the object in the image space and the best behavior is obtained when **W** = **M**^−2^. The 3D trajectories described by the manipulated object are shown in Figure 8. This last figure only represents the face of the manipulated object observed by the eye-to-hand camera. The desired 3D trajectory is indicated by white rectangles and the desired 3D positions of the extracted features are indicated in blue. The robot is performing a desplacement in the Y axis direction while the manipulation does a rotation of the object. As in the previous cases, the 3D trajectories obtained when **W** = **M**^−2^, **W** = **DM**^−2^ and **W** = **M**^−1^ are indicated in green, red and orange respectively. As it is shown in Figure 8, a correct 3D behaviour is observed and the manipulation is correctly developed by using the proposed visual controllers. In this case, lower errors are obtained setting **W** = **M**^−2^.

**Figure 9.**
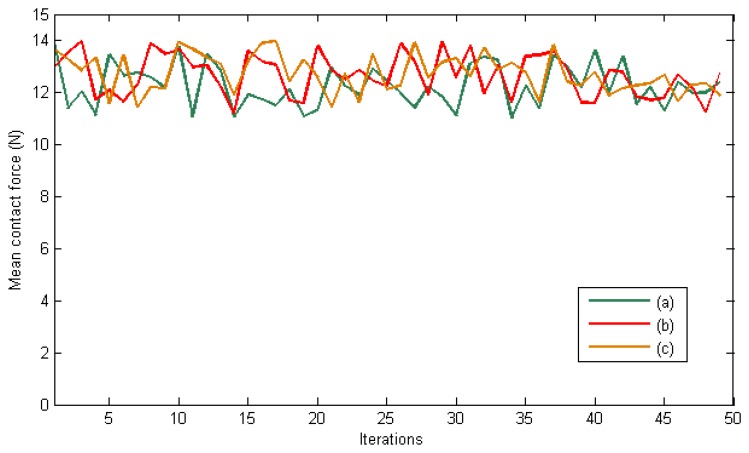
Mean total contact force during the second set of experiments. (**a**) **W** = **M**^−2^. (**b**) **W** = **DM**^−2^. (c) **W** = **M**^−1^.

**Figure 10.**
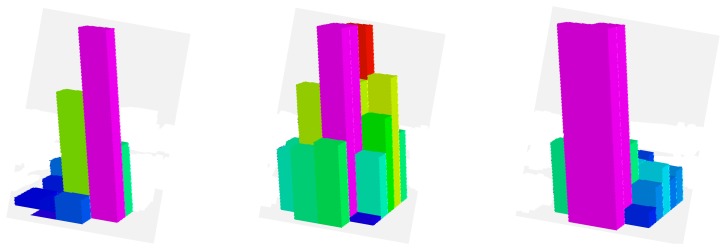
Distribution of the pressure measurements registered by the arrays of tactile sensors of the three fingers in an intermediate iteration during the manipulation task (second task).

Finally, Figures 9 and 10 represent the evolution of the mean contact force and the distribuition of the pressure over the tactile sensors respectivley. As it can be seen, the desired contact force in this case is regulated to 12.5 N. Although the proposed visual controllers have shown a correct behavior to guide a robotic hand during manipulation tasks, the proposed framework can be employed to define new ones by modifying the value of the matrix **W**.

## 9. Conclusions

This paper presents a novel optimal control framework which allows defining new dynamic visual controllers in order to carry out the dexterous manipulation of a robotic manipulation system. Making use of both the dynamics of the robotic hand and the definition of the image trajectory as task description of the object motion, different image-based dynamic visual servoing systems are defined to dexterous manipulation with multi-fingered robotic hands. Force fingertip control has been proposed as an additional control command which acts in the null-space of the task which manages the object tracking. This way, the desired contact force can be set independently and accomplished with any of the control laws derived from optimal control framework. For that end, a set of tactile sensors has been used in the real experiments in order to verify the proposed control law.

The approach has been successfully verified with the implementation of some derived controllers on a real robotic manipulation system. As shown, the behavior in task space is very similar and the image error remains low using different values of the weighting matrix. The fingertip interaction force is also regulated and low error is obtained during the manipulation tasks.

## Figures and Tables

**Table 1.** Mean image and force contact error during the first set of experiments.

**W**	**Image Error (Px)**	**Force Contact Error (N)**
**M**^−2^	2.487	0.87
**DM**^−2^	2.621	1.01
**M**^−1^	4.010	0.91
